# Pancreatic hepatoid adenocarcinoma with neuroendocrine differentiation and elevated AFP: a case report

**DOI:** 10.3389/fonc.2025.1652925

**Published:** 2025-08-18

**Authors:** Zhouliang Yang, Ting Li, Xiaowei Zhang

**Affiliations:** ^1^ Department of Pathology, The First People’s Hospital of Yongkang, Yongkang, Zhejiang, China; ^2^ Department of Imaging Medicine, The First People’s Hospital of Yongkang, Yongkang, Zhejiang, China; ^3^ Department of Pathology, Affiliated Dongyang Hospital of Wenzhou Medical University, Dongyang, Zhejiang, China

**Keywords:** hepatocellular adenocarcinoma, pancreatic cancer, neuroendocrine differentiation, alpha-fetoprotein, rare tumors, surgical resection

## Abstract

**Background:**

Hepatoid adenocarcinoma (HAC) of the pancreas is a rare malignant tumor characterized by morphologic and immunophenotypic features resembling hepatocellular carcinoma. The tumor cells exhibit polygonal morphology with eosinophilic or clear cytoplasm and large, irregular nuclei. Immunophenotypically, the tumor cells are positive for alpha-fetoprotein (AFP) and glypican-3. Pancreatic HAC is rare, and neuroendocrine differentiation often complicates both diagnosis and treatment.

**Case description:**

We report a case of pancreatic HAC with neuroendocrine differentiation and elevated AFP levels. A 46-year-old man was hospitalized due to progressive jaundice and dark urine that persisted for a week. Physical examination revealed bile duct dilation, a pancreatic head mass, and markedly increased AFP levels. The patient, with a history of schizophrenia and lung cancer surgery 2 years prior, was on regular medication. Following a pancreaticoduodenectomy, postoperative monitoring indicated normalization of AFP levels.

**Conclusion:**

Elevated serum AFP levels may be a crucial indicator for preoperative pancreatic HAC diagnosis. Additionally, pancreatic HAC has unique histological and immunophenotypic characteristics. However, neuroendocrine differentiation complicates diagnosis and treatment. Therefore, complete surgical resection is the optimal treatment option for this condition.

## 1 Introduction

Hepatoid adenocarcinoma (HAC) is an extrahepatic malignant tumor that resembles hepatocellular carcinoma clinically and pathologically. The tumor commonly originates in the stomach but may affect other organs such as the lungs, bladder, kidney, uterus, fallopian tubes, and ovaries. Furthermore, HAC is exceptionally rare in the pancreas. Pancreatic HAC with neuroendocrine differentiation is a rare malignant tumor that exhibits both the histological features of HAC and neuroendocrine differentiation. Owing to its low incidence rate and the high likelihood of being overlooked or misdiagnosed, pancreatic HAC with neuroendocrine differentiation poses a critical challenge in clinical diagnosis and treatment. Herein, we present a case of pancreatic HAC with neuroendocrine differentiation and elevated AFP treated at our hospital. This report aims to provide insights into the diagnosis and treatment of pancreatic HAC.

## 2 Case presentation

A 46-year-old male farmer was hospitalized on January 6, 2025, due to progressive jaundice of the skin and sclera, accompanied by dark urine that had persisted for a week. He reported decreased appetite and weakness without nausea, vomiting, chills, fever, or abdominal pain. Physical examination revealed jaundice of the skin and sclera and a tender abdomen, but no significant rebound tenderness, palpable masses, or abdominal distention. The liver and spleen were not palpable below the costal margin. Laboratory tests showed an increased AFP level of 338.53 ng/mL, carbohydrate antigen 19-9 (CA 19-9) level of 40.1 U/mL, normal carcinoembryonic antigen (CEA) level, alanine aminotransferase (ALT) level of 88 U/L, and aspartate aminotransferase (AST) level of 20 U/L. The patient’s mental state was normal during this hospitalization.

### 2.1 Imaging examinations

#### 2.1.1 Magnetic resonance cholangiopancreatography

The intrahepatic and extrahepatic bile ducts were dilated, with a small oval filling defect observed in the lower segment of the common bile duct. Both the gallbladder and the pancreatic duct were normal.

The diagnosis revealed dilation of the intrahepatic and extrahepatic bile ducts with potential stones in the lower segment of the common bile duct. Incidentally, a small amount of fluid was detected around the spleen.

#### 2.1.2 Computed tomography

There was no hepatomegaly. Dilation of the intrahepatic and extrahepatic bile ducts was observed. The wall of the lower end of the common bile duct was thickened and exhibited ring enhancement. The gallbladder was enlarged with smooth walls without thickening. Splenomegaly was indicated. The pancreas and bowel walls were normal. Additionally, no ascites or enlarged retroperitoneal lymph nodes were reported.

The diagnosis revealed mild thickening and enhancement at the distal end of the common bile duct, accompanied by dilation of the intrahepatic and extrahepatic bile ducts. Therefore, further examination was recommended.

#### 2.1.3 Magnetic resonance imaging

**Figure 1:**
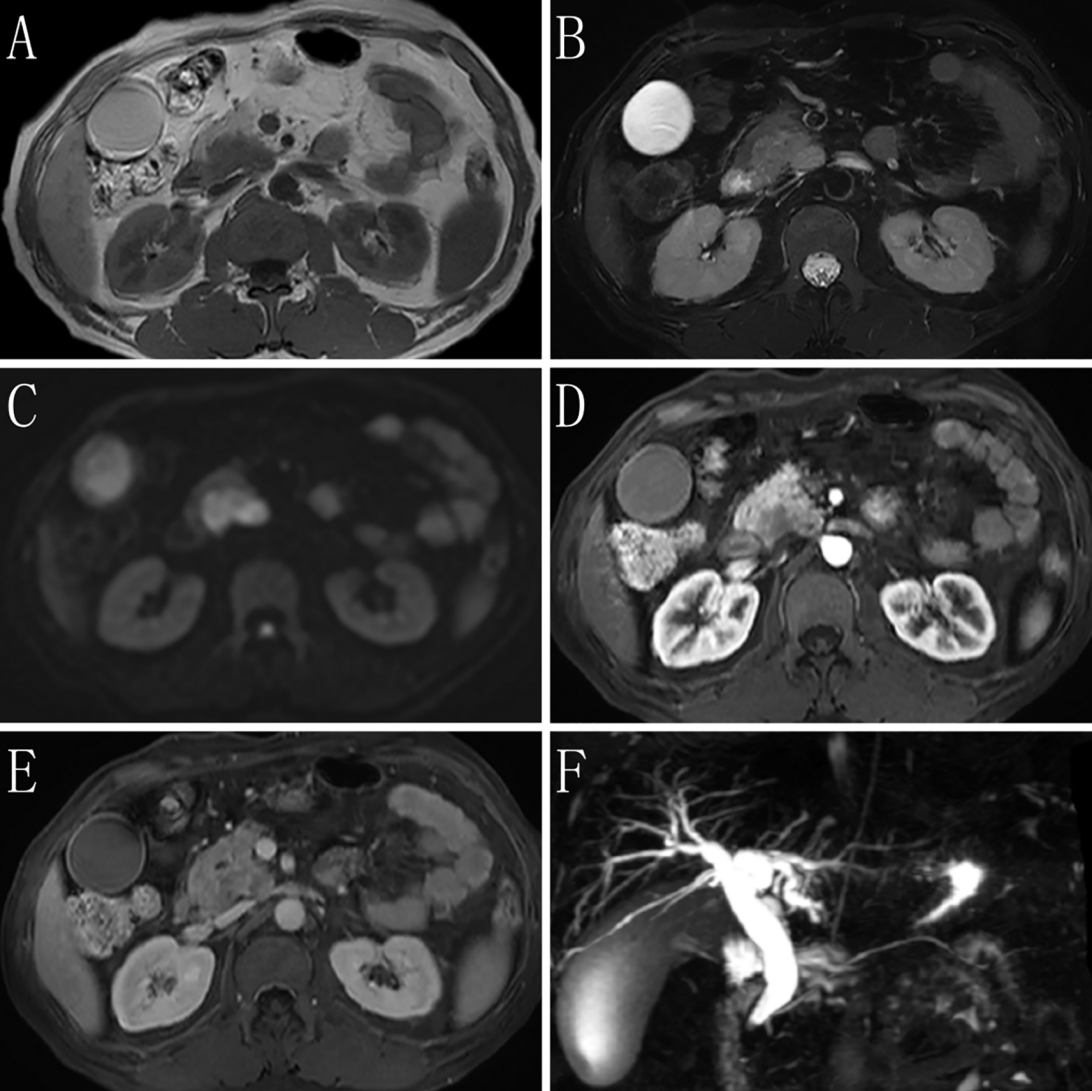
Magnetic resonance imaging (MRI) findings of the case. **(A)** T1-weighted imaging (T1WI) shows the lesion having a slightly low signal. **(B)** T2-weighted imaging (T2WI) shows most of the lesion having slightly high signal on fat-suppressed transverse sections. The lesion has clear boundaries, and fibrous septa are visible within it. Enlarged lymph nodes are seen in the surrounding area. **(C)** Diffusion-weighted imaging (DWI) shows that the tumor parenchyma has a high signal. **(D)** The arterial phase of the enhanced imaging shows that the lesion has a low signal, with no involvement of surrounding blood vessels or organs. **(E)** The delayed phase of the enhanced imaging shows progressive enhancement of the lesion, which is lower than that of the surrounding normal pancreatic parenchyma. Low-signal septa within the lesion and a surrounding ring-like enhancing capsule are observed. **(F)** Magnetic resonance cholangiopancreatography (MRCP) shows dilation of the bile duct, with no dilation of the pancreatic duct.

MRI images are shown in **Figure 1**. No liver enlargement was observed, and the intrahepatic and extrahepatic bile ducts were dilated. The lower end of the common bile duct appeared narrowed or invaded. A mass-like abnormal signal, measuring approximately 40 × 25 mm, was detected in the pancreatic head. This mass exhibited a slightly high signal on T2-weighted imaging, high signal intensity with restricted diffusion on diffusion-weighted imaging, a reduced apparent diffusion coefficient, and a region of low enhancement on the contrast-enhanced scan with indistinct boundaries. The gallbladder appeared distended and dilated, featuring a smooth wall without thickening. Additionally, the spleen was enlarged, with no abnormal signals noted.

Imaging suggested that the pancreatic head mass was suspicious for a tumor, causing compression or invasion of the lower end of the common bile duct, resulting in bile duct dilation. Splenomegaly was also observed.

### 2.2 Medical history

The patient had a history of schizophrenia for over 15 years and was on medications regularly. He also had a history of early-stage lung cancer surgery 2 years prior to presentation. He had no prior history of tumors, and no known family history of hereditary diseases.

### 2.3 Surgical procedure and pathology

**Figure 2:**
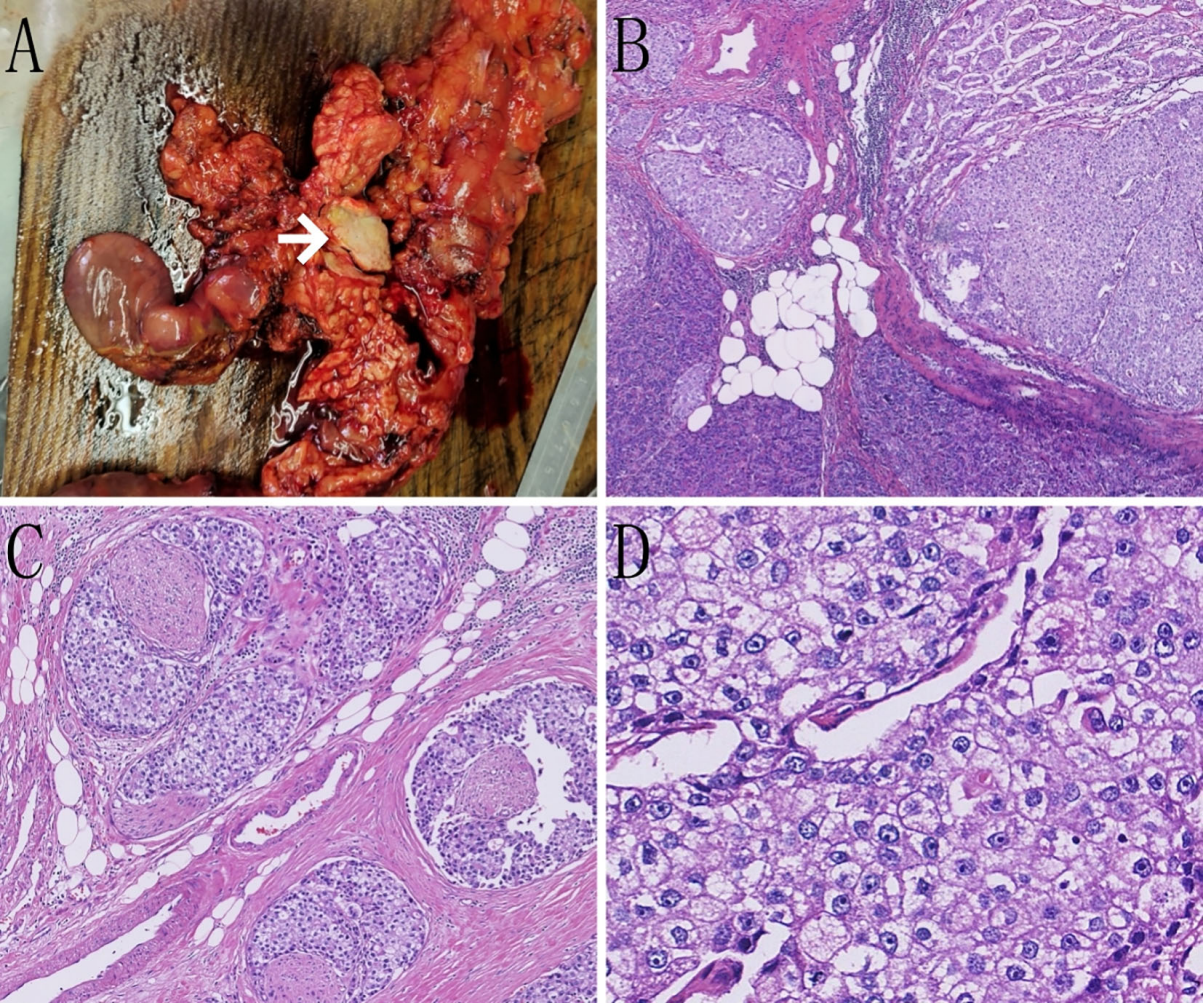
Pathological images of the case. **(A)** On gross examination, a grayish-white, hard mass is observed within the pancreatic tissue (indicated by the white arrow). **(B)** Tumor cells are seen infiltrating the surrounding pancreatic tissue in nests and clusters (hematoxylin and eosin staining [H&E], ×50). **(C)** Tumor cells are seen infiltrating the nerves in nests and clusters (H&E, ×100). **(D)** Tumor cells display an eosinophilic or clear cytoplasm, with round or irregular nuclei and prominent nucleoli (H&E, ×400).

On January 12, 2025, the patient underwent laparoscopic pancreaticoduodenectomy. Intraoperatively, a 3 × 2 cm mass was identified in the head of the pancreas. On cut section, it was gray-white, firm, and poorly circumscribed. No enlarged peripancreatic lymph nodes were seen. The tumor cells exhibited a nesting and lobular arrangement with small tubular structures. The stroma displayed high vascularity. The cytoplasm appeared eosinophilic or clear, whereas the nuclei were round or irregular, with thick nuclear membranes and prominent nucleoli. Additionally, small areas of apoptotic and necrotic cells were present. The tumor exhibited infiltrative growth, involving nerves and the surrounding pancreatic tissue, and displaying cancer emboli in blood vessels (**Figure 2**).

**Figure 3:**
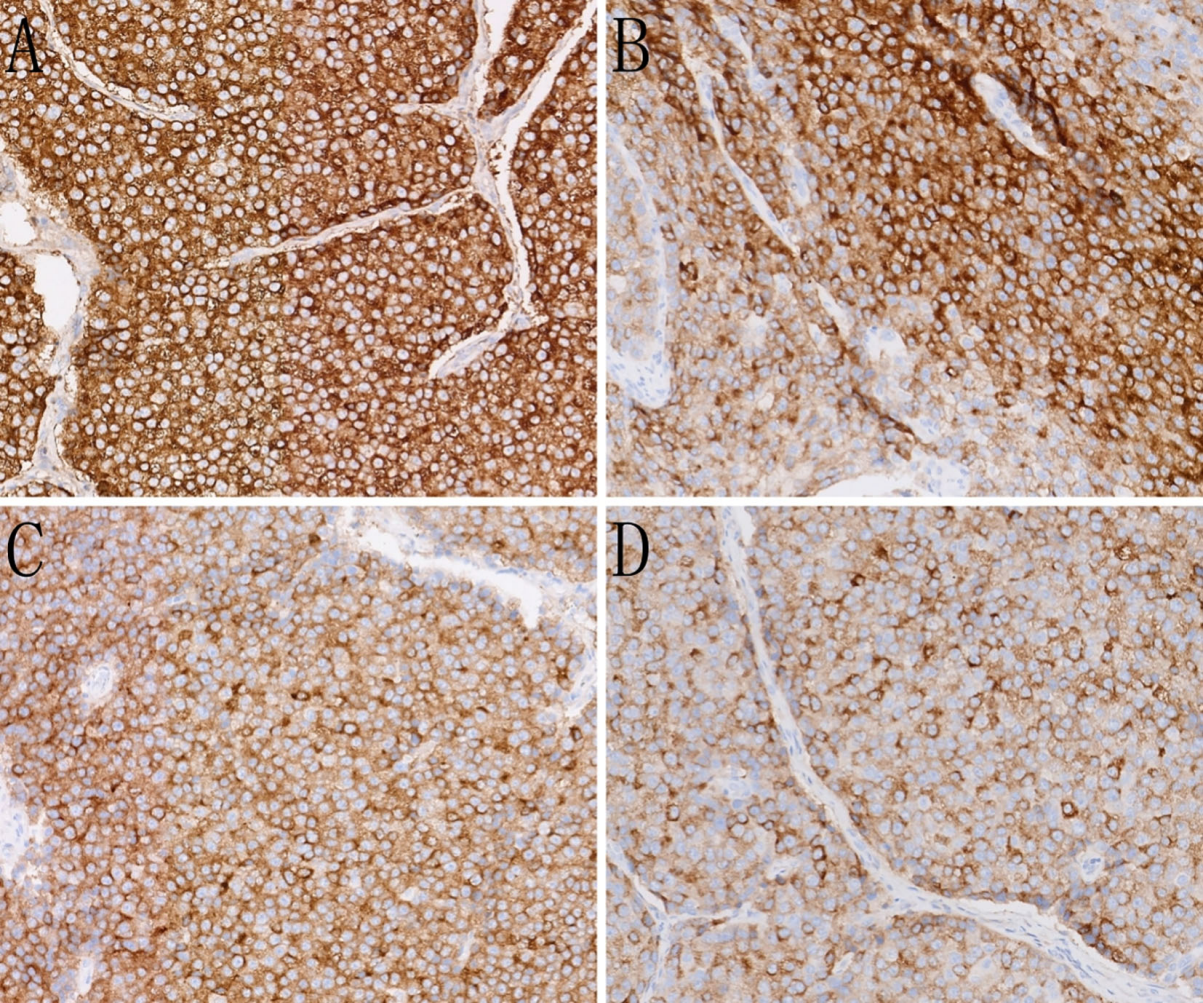
Immunohistochemistry (IHC) of the case. **(A)** The tumor cells show strong expression of alpha-fetoprotein (AFP)(IHC, ×200). **(B)** The tumor cells show strong expression of glypican-3 (IHC, ×200). **(C)** The tumor cells show strong expression of the neuroendocrine marker SYN (IHC, ×200). **(D)** The tumor cells show strong expression of the neuroendocrine marker CGA (IHC, ×200).

Immunohistochemistry results are shown in **Figure 3**. Evaluation of neuroendocrine markers revealed that the tumor cells were positive for creatine kinase and chromogranin A (CgA), synaptophysin (Syn), and insulinoma-associated protein 1, and negative for cluster of differentiation (CD) 56. Assessment of HAC markers revealed that the tumor cells were positive for AFP and glypican-3; negative for Sal-like protein 4; strongly positive for human epidermal growth factor receptor 2 (3+); positive for Brahma-related gene 1 and retinoblastoma 1; and negative for CD117 and S100.

Pathological diagnosis: The morphology and immunohistochemistry of the pancreatic head tissue were consistent with HAC with neuroendocrine differentiation, elevated AFP, vascular tumor thrombus, and neural invasion. The margins of the stomach, duodenum, common bile duct, and pancreas were negative. Histopathology revealed chronic cholecystitis. Among the examined lymph nodes, one of the seven peripancreatic lymph nodes, none of the four mesenteric lymph nodes, and none of the four perigastric lymph nodes showed cancer metastasis. According to the 2017 AJCC on Cancer staging system, the tumor stage was pT2N1Mx.

### 2.4 Postoperative follow-up

The patient was hospitalized for 21 days, during which primary wound healing was achieved and no surgical complications occurred. Two weeks postoperatively, laboratory test results were as follows: CEA, 2.18 ng/mL; AFP, 2.86 ng/mL; ALT, 16 U/L; and AST, 17 U/L. Postoperative chemotherapy and radiation therapy were declined by the family due to concerns about the patient’s psychiatric condition. A follow-up assessment conducted 4 months post-surgery revealed no signs of recurrence or distant metastasis.

## 3 Discussion

HAC manifests in various organs, including the esophagus, stomach, gallbladder, colorectum, lungs, and ovaries (1). However, its occurrence in the pancreas is uncommon, with pancreatic HAC showing greater rarity, particularly with neuroendocrine differentiation. The precise histological origin of pancreatic HAC remains unclear; however, two primary mechanisms of its occurrence are proposed. First, both the pancreas and liver derive from the primitive foregut during embryonic development. During early embryogenesis, certain cells may form tumors exhibiting liver-like characteristics due to aberrant differentiation (2). Secondly, pancreatic acinar cells undergo transdifferentiation into hepatocytes under specific conditions, such as gene mutations or chronic inflammation (3).

Imaging studies, such as pancreatic CT and MRI, typically lack specificity but are crucial for determining the location, size, invasion extent, and presence of metastasis. HAC typically presents as a solitary, relatively large, solid mass with well-defined margins. On contrast-enhanced imaging, it shows mild-to-moderate arterial-phase enhancement followed by washout in the portal-venous or delayed phases—similar to the “rapid-in/rapid-out” pattern of hepatocellular carcinoma. However, the degree of enhancement is generally lower than that seen in hepatocellular carcinoma. MRI features include a slightly high signal on T2-weighted imaging and moderate progressive enhancement on contrast-enhanced scans. These findings suggest a combination of hepatocellular components and neuroendocrine differentiation. Histological assessment serves as the gold standard for diagnosing pancreatic HAC. The tumor cells exhibit expansive infiltrative growth and a morphology resembling that of hepatocellular carcinoma, primarily comprising polygonal cells with eosinophilic cytoplasm arranged in trabecular and nested patterns. Pancreatic HAC is categorized into four histological subtypes: 1) pure hepatocellular carcinoma-like morphology, 2) neuroendocrine morphology, 3) glandular morphology, and 4) acinar cell morphology (4). Immunohistochemical detection is pivotal for diagnosis, with the combined use of hepatocellular markers (e.g., AFP, hepatocyte paraffin-1, and glypican-3) and neuroendocrine markers (e.g., CgA, Syn, and CGA) aiding in accurate diagnosis. Owing to the similarities between pancreatic HAC and metastatic hepatocellular carcinoma in terms of morphology, immunohistochemistry, and AFP expression, the primary differential diagnosis is metastatic hepatocellular carcinoma. The patient’s immunohistochemistry aligned with a blend of HAC-like morphology and neuroendocrine morphology. Furthermore, the absence of a definitive hepatic mass on preoperative imaging studies and postoperative examinations confirmed the diagnosis of pancreatic HAC with neuroendocrine differentiation.

Patients with pancreatic HAC may exhibit elevated serum AFP levels, which hold preoperative diagnostic and prognostic significance (5). AFP serves as a prognostic indicator in patients with HAC. Following treatment, AFP levels typically decrease in these patients (6). However, as AFP is not a specific marker for this tumor, the absence of AFP overexpression does not exclude a diagnosis of HAC (7, 8). In this case, the patient initially presented with elevated AFP levels in the blood. Subsequent postoperative monitoring revealed a normalization of AFP levels, suggesting a close association between AFP and the tumor.

Pancreatic HAC with neuroendocrine differentiation typically carries a poor prognosis. Factors influencing tumor outlook include tumor size, vascular invasion, liver metastasis, and lymph node metastasis. Additionally, elevated preoperative AFP levels may indicate an unfavorable prognosis (9). Surgical resection remains the primary treatment for this tumor variant, significantly improving survival rates. However, due to the tumor’s high aggressiveness and frequent vascular, liver, or lymph node metastasis, preoperative diagnosis is challenging, and many patients lose the opportunity for curative surgery during the diagnostic process (10, 11). Studies indicate that surgery is the most common treatment for localized pancreatic HAC and improves survival, with a median overall survival of 12.5–13 months, a 1-year survival rate of 71%, and a 2-year survival rate of 40% (12, 13).

Chemotherapy may be effective in some patients; however, the overall prognosis remains unsatisfactory. Therefore, targeted therapy and immunotherapy may offer new treatment hopes for these patients (14).

The limitations of this case report include the absence of genetic or molecular analyses and a short follow-up period of only 4 months.

In conclusion, pancreatic HAC with neuroendocrine differentiation is an extremely rare subtype of pancreatic cancer that lacks distinctive clinical characteristics. The absence of hepatitis of infectious etiology, high serum AFP levels, and the presence of a pancreatic mass on imaging may indicate the presence of pancreatic HAC. Diagnosis primarily depends on pathological findings, and the positive expression of hepatocellular carcinoma markers using immunohistochemistry aids in diagnosis. Taken together, radical surgical resection seems to be the preferred treatment, and thorough postoperative management may provide relief to patients.

## 4 Patient perspective

“The surgery was a difficult experience, but I felt reassured by my medical team. Recovery took time, but I am grateful for the support I received. I have learned to appreciate my health more and encourage others to seek medical attention early if they notice unusual symptoms”.

## Data Availability

The original contributions presented in the study are included in the article/supplementary material. Further inquiries can be directed to the corresponding authors.
